# Improving response rate and quality of survey data with a scratch lottery ticket incentive

**DOI:** 10.1186/1471-2288-12-52

**Published:** 2012-04-19

**Authors:** Frank Olsen, Birgit Abelsen, Jan Abel. Olsen

**Affiliations:** 1 Institute of Community Medicine, Faculty of Health Sciences, University of Tromsø, N-9037, Tromsø, Norway; 2 Center of Clinical Documentation and Evaluation, Regional Health Authority of Northern Norway, PB 6, N-9038, Tromsø, Norway; 3 Northern Research Institute Alta, PB 1463, N-9506, Alta, Norway

**Keywords:** Incentive, Response rate, Survey, Nonresponse error, Item nonresponse

## Abstract

**Background:**

The quality of data collected in survey research is usually indicated by the response rate; the representativeness of the sample, and; the rate of completed questions (item-response). In attempting to improve a generally declining response rate in surveys considerable efforts are being made through follow-up mailings and various types of incentives. This study examines effects of including a scratch lottery ticket in the invitation letter to a survey.

**Method:**

Questionnaires concerning oral health were mailed to a random sample of 2,400 adults. A systematically selected half of the sample (1,200 adults) received a questionnaire including a scratch lottery ticket. One reminder without the incentive was sent.

**Results:**

The incentive increased the response rate and improved representativeness by reaching more respondents with lower education. Furthermore, it reduced item nonresponse. The initial incentive had no effect on the propensity to respond after the reminder.

**Conclusion:**

When attempting to improve survey data, three issues become important: response rate, representativeness, and item-response. This study shows that including a scratch lottery ticket in the invitation letter performs well on all the three.

## Background

Although there seems to be general agreement that good survey practice requires high response rates, Groves [1] shows that there is little empirical support for the notion that low response rates *de facto* produce estimates with high nonresponse bias. Still the quality of data collected in survey research is often indicated by the response rate; the representativeness of the sample, and; the rate of completed questions (item-response). In attempting to improve a generally declining response rate in surveys considerable efforts are being made through follow-up mailings and various types of incentives [2-6]. Reminders are the most common method for improving response rates, but the use of monetary and non-monetary incentives have a long history as methods of improving response rates [7-10]. The aim of this paper is to study the effects on response rate, sampling error, and item response, of including a scratch lottery ticket incentive in the invitation letter to a survey. Previous studies suggest that, in general, incentives work. Monetary incentives - especially cash - are more effective than non-monetary incentives, and prepaid incentives are more effective than conditional ones [11-14]. There is also evidence suggesting increasing response rates with increasing value of prepaid monetary incentives [15].

A general problem in mail surveys is underrepresentation of lower socio-demographic groups. Some studies indicate that incentives may improve representativeness because they have relatively higher effect in those socio-demographic groups with a relatively lower response rate [7,10,16].

Concern has been raised that incentives might reduce data quality, but there appears to be little empirical evidence supporting this [7]. To the contrary, some studies indicate that incentives may influence respondents to put more efforts into completing all questions included in the questionnaire [12,16].

The research on the effects of using lottery incentives to increase survey response rates is meagre and equivocal [11,17-25]. Some studies report increased response rates due to lottery incentives [19-21], while others report no significant effects [17,18,22-25]. The type of lottery incentive seems to be of importance. Instant lotteries like scratch lotteries appear to be more effective than the type where respondents are entered into lottery style draws put up for a particular survey, or the type where lottery tickets are rewarded conditional on response. Deutskens et al. [26] show that lotteries are efficient in short surveys, and that lotteries with small prizes but a high chance of winning are most effective in increasing the response rate. Göritz [27] reports no lottery incentive effect on response quality, while Bonke and Fallesen [28] reports the opposite.

A recent Cochrane Review [11] identified 94 trials of various non-monetary incentive (e.g. key ring, lottery participation) and found the odds of response increased by over a tenth when non-monetary incentives had been included (OR 1.15, 95% CI 1.08 to 1.22). Out of the 23 studies that had utilized some form of lottery incentive nine demonstrated a significant benefit from lottery incentive [19-21,29-34]. However, the effectiveness depends on whether the lottery ticket had been conditional or not. The lottery ticket was conditional in 17 of the 23 studies, only five of which demonstrated significant effects on response rate [21,29-32]. The remaining six studies used unconditional lottery tickets, in four of which the lottery incentive demonstrated significant increased response rates [19,20,33,34].

### Research setting and aim

Effective alternatives to monetary incentives are often requested because postal authorities in many countries dissuade sending cash in the mail [18]. Furthermore, experience from Norway indicates that some recipients might be offended by being offered money to participate in scientific research [33]. Because of a relatively higher demand for scratch lottery tickets in low income and low educational groups compared to high income and high educational groups in Norway [35], it was hypothesised that this particular incentive would evoke relatively more positive attitude among people in this group, and hence increase their propensity to participate.

This paper inquire into the following: First, to which extent does a scratch lottery incentive increase survey response? Second, to which extent does this incentive improve the quality of the data in terms of representativeness and item nonresponse? Third, does the incentive included in a *first wave*, *i.e.* together with the invitation letter and questionnaire, have any remaining incentive effect in a *second wave* where non-responders receive a reminder that does not include a scratch lottery ticket?

## Methods

### Questionnaire

The questionnaire included 14 questions: nine related to oral health and five to socio-demographic characteristics such as: education (primary school, secondary school or university level); main source of income; total household income; household size and composition. The questions asked either for one multi-optional answer, or an open answer.

### Sample

The questionnaire was mailed to random population samples in Norway: 800 adults (aged 21 – 60) in each of three counties, giving a total sample of 2,400. The counties were chosen because of their differences in dentist densities, while the particular age group was chosen because they pay all costs for dental services out of pocket. The samples were randomly drawn from the Norwegian Population Register, and included information on age, gender, and home address (postal code). The survey was reported to the Privacy Ombudsman for Research in Norway in accordance with notification requirements. It also conforms to the ethical principles of the Helsinki Declaration. Approval from any ethics committee was not necessary for this survey.

### Survey and experimental design

A systematically selected half (every second person on the list) in each county sample received a scratch lottery ticket costing 3 EUR together with the questionnaire, while the other half received the questionnaire only, *i.e.* 1,200 in the incentive group and 1,200 in the control group.

The scratch lottery ticket used is a continuously run national lottery that most Norwegians are familiar with. The lottery was introduced in 1988 by a government owned games company (Norsk Tipping). It is well known and highly trusted . Scraping the coating on the front of these tickets reveals if you win money or not. The lottery consists of 6 million tickets, of which 1.5 million tickets give a prize, *i.e.* the probability of winning is 0.25. Information about the high probability of winning has been proclaimed in numerous TV and newspapers commercials for several years. From the total revenues, 50% is returned in terms of winning prizes, the first prize is approximately 120,000 EUR. The most common prize is the same as the cost of the lottery ticket. This national lottery is the largest scratch lottery in Europe measured by purchase per capita. On average every Norwegian buys seven scratch lottery tickets each year in this lottery. The scale of the lottery and its long history indicate that most people should be sufficiently aware of the high probability of winning.

This chosen incentive was unconditional in that respondents were informed to consider the scratch lottery ticket as a gift whether or not they responded to the questionnaire. The only piece of information differing in the letter of invitation between the incentive group versus the control group was this sentence: ‘As an expression of our gratitude, we are happy to enclose a scratch lottery ticket as a gift. The gift is yours whether or not you respond to the questionnaire’*.* Six weeks after, a reminder-questionnaire *without* incentives was mailed to non-respondents in both groups.

The data were analysed using one-sample and two-sample t-tests, chi-square test, logistic regression analysis and univariate generalized linear model analysis.

## Results

Table 1 presents the sample, the pre-reminder response rate, and the total response rate in the incentive and control groups. There were 94 withdraws (40 in the incentive group and 54 in the control group) due to addressee’s death, unknown address or lack of capability of answering the questionnaire due to self-reported illness.

**Table 1:** Sample, response, and response rates

	**Incentive group**	**Control group**
**Sample**	1 200	1 200
**Sample after withdraws**	1 161	1 145
**Pre-reminder response**	558	443
**Pre-reminder response rate**^**1**^	48.1%	38.7%
**Reminder response**	187	229
**Reminder response rate**^**1**^	187/603 = 31.0%	229/702 = 32.6%
**Total response**	745	672
**Total response rate**^**1**^	64.2%	58.7%

^1^ Response rate after withdraws.

Independent samples t-tests with incentive/no-incentive as grouping variable, and gender and age as test variables showed no statistically significant differences between the incentive group sample and the control group sample (p-values were 0.308 and 0.136, respectively).

### Did the incentive increase the response rate?

The pre-reminder response rate was 48.1% in the incentive group compared to 38.7% in the control group. After the reminder, the relative difference in response rates was smaller; 64.2% in the incentive group vs 58.7% in the control group.

To test whether or not there was significant differences in the response rates in the two groups logistic regression analysis was performed. Table 2 shows that the lottery ticket incentive had significantly positive effects on the response rate, both before and after the reminder.

**Table 2:** Logistic regression, effect on response rate, incentive vs. control group

	**Oddsratio**	**p-value**	**95% CI**	**Parameter estimate**	**S.E**
**Pre-reminder response**	1.466	< 0.0001	1.243 – 1.730	0.383	0.084
**Total response**	1.261	0.007	1.066 – 1.491	0.232	0.086

Pre-reminder response and total response (separate logistic equations).

There was no confounding with other variables, like gender, age and county of residence. We assumed that a smaller change than 10% in the incentive odds-ratio means there is no confounding. When including gender, age and county of residence as explanatory variables in the logistic regression models, this gave only small changes in the incentive odds-ratios. Hence, after adjusting for other variables the incentive still had a statistically significant positive effect on the response rate.

### Did the incentive reduce nonresponse error?

To test the representativeness of the respondents we compared the gender balance, mean age and mean education level (primary school = 1, other education level = 0) among the respondents in the incentive vs control groups with the population means (aged 21–60), using one-sample t-tests. Both groups differed from the population means in that they included a significantly higher share of women, had a significantly higher mean age, and people with a significantly higher mean education level (both p-values < 0.001). Both respondent groups were in other words biased, but the incentive respondents appeared to be *less* biased.

To test if the incentive respondents were *significantly* less biased than the control group respondents, independent sample t-tests were used to compare the gender balance, mean age, and mean educational level across the respondent groups. Education level was the only variable that differed significantly (see p-values in Table 3), when comparing the incentive respondents with the control respondents, both before the reminder and after the reminder. This indicates that the incentive increased the share of less educated people, *i.e.* reduced nonresponse error.

**Table 3:** Population, incentive and control group respondent characteristics

**Variabels**	**Population (aged 21–60)**	**Pre-reminder**			**Total**		
		**Mean**		**p-value**	**Mean**		**p-value**
		**Incentive group**	**Control group**	**Incentive vs control group**	**Incentive group**	**Control group**	**incentive vs control group**
**Gender**	1.51	1.44	1.39	0.174	1.47	1.43	0.160
**Age**	40.89	42.69	43.37	0.313	42.61	42.88	0.643
**Education level**^**2**^	0.23	0.15	0.10	0.024	0.15	0.11	0.015

^1^ Gender: 1 = woman, 2 = man. ^2^ Education level: 1 = primary school, 0 = other.

### Did the incentive increase item response?

The questionnaire contained nine questions about oral health. Table 4 shows the mean number of questions answered and the results from tests of difference in means by regression analysis performed to study the effect of the incentive on the item response. A general linear regression model was used to test the difference in means between the incentive and control group. The incentive group answered a significantly higher mean number of questions than the control group, both before and after the reminder, and also when controlled for demographic characteristics. Age and education had a significantly positive effect on the number of questions answered both in the incentive group and the control group, both before and after the reminder.

**Table 4:** Number of questions answered

	Model 1*		
	**Mean (S.E)**		**p-value**
	**Incentive group**	**Control group**	**Incentive vs control group**
**a) Pre-reminder**	6.72 (0.033)	6.60 (0.038)	0.015
**b) Total**	6.70 (0.030)	6.56 (0.032)	0.001
	Model 2**		
	**Mean (S.E)**		**p-value**
	**Incentive group**	**Control group**	**Incentive vs control group**
**a) Pre-reminder**	6.71 (0.032)	6.60 (0.036)	0.029
**b) Total**	6.70 (0.029)	6.57 (0.031)	0.002

GLM Univariate Analysis.

* Model 1. Number of questions answered as dependent variable and respondents in the incentive group or controll group (1,0) as fixed factor.

** Model 2. The same as model 1 but with gender, age and education as covariates. Age and education are significant in both the pre-reminder and total models.

### Did the incentive affect the propensity to respond after the reminder?

The incentive was given with the invitation letter only. In order to test for any incentive effect after the reminder, a logistic regression analysis included those who responded after the reminder (total 416; 187 with incentive, 229 without), and those who did not respond at all (total 889; 415 with incentive, 474 without). Response (no/yes) was the dependent variable, and incentive (no/yes) was the independent variable. An odds-ratio of 0.920 (p = 0.482, 95% CI = [0.728 – 1.162]), means no significant effect of the incentive on increasing the response rate after the reminder.

### Is a scratch lottery ticket a cost effective incentive?

We define the most cost effective way of increasing the response rate as the procedure that gives the lowest *cost pr additional respondent*. For the incentive group prior to the reminder the cost was 3,600 EUR (1,200 scratch lottery tickets times 3 EUR). The number of additional respondents was calculated as the number of incentive group respondents who responded before the reminder (558), subtracted by the control group respondents who responded before the reminder (443), which gave 115 additional respondents. Hence costs per additional respondent was 3,600/115 = 31 EUR.

For the control group the cost of the reminder was estimated to 3,468 EUR. This includes administrative costs (two minutes additional work for each of the 702 reminders at the labour cost of 60 EUR pr hour; 1,404 EUR), plus sheets, printing, stamps and envelopes costs (3 EUR for each reminder). The reminder gave 229 additional respondents in the control group. Hence, the cost per additional respondent was 3,468/229 = 15 EUR, *i.e.* in this survey the reminder was a cheaper way to increase the response rate than the scratch lottery ticket. We also carried out a sensitivity analysis on the cost per additional respondent when varying the time spent on administrating the reminder. Three and five minutes gave a cost per additional respondent of respectively 18 EUR and 24 EUR, *i.e.* the reminder was still cheaper than the scratch lottery ticket.

## Discussion

This study shows that a lottery ticket incentive may have *three* important positive effects on data collection in survey research. First, it improves the response rate. The scratch lottery ticket incentive used in this study gave a higher odds ratio than the average for the 94 studies of non-monetary incentives identified in the recent Cochrane Review [11].

Second, our chosen incentive improved representativeness. A common problem with sample surveys is that lower socio-demographic groups are less likely to respond. The scratch lottery ticket gave a significantly more representative sample in terms of education level. Groves [1] finds that covariance between survey variables and response propensities are highly variable across items within a survey, survey conditions, and populations. However this study gives us reason to conclude as proposed by Singer et al. [9] and Martin, Abreu and Winters [36] that using incentives can reduce nonresponse bias by increasing the number of respondents who are often underrepresented, especially those with the lowest education, even if the scratch lottery ticket did not completely solve the sampling error problem.

Third, respondents who got a scratch lottery ticket completed more questions. The quality of the data in terms of item response was as expected, significantly in favour of the incentive group, something which correspond with earlier findings by Willimack et al. [37] and James and Bolstein [16]. Such increased efforts among respondents to complete questionnaires might be explained by Gouldner’s [38] norm of reciprocity claiming the existence of a social normative standard, leading individuals to strive to repay favours freely given.

The success of including a scratch lottery ticket in this study could be due to a combination of several factors: a balanced connection between the size of an unconditional gift and the effort needed to complete the relatively short questionnaire; a trusted lottery organised by a well- known government owned games company; a well known high probability of winning and a well known and relatively high expected value, and finally; it was an instant lottery rather than an internal future prize drawn up by survey researchers.

## Conclusion

The results of this study support previous findings that an incentive improves the response rate, as well as the representativeness of the sample. Research grants are limited, and most of us face a challenge collecting the best possible data given a survey budget. Although comparing the cost per additional respondent suggest that a reminder would be cheaper than the scratch lottery tickets, it is important also to compare representativeness as well as item-response when choosing between alternative ways of increasing survey data quantity and quality.

## Competing interests

The authors declare that they have no competing interests.

## Authors’ contribution

All authors contributed to designing the survey, interpreting the results, drafting and revising the manuscript, read and approve the final manuscript. FO and BA analyzed the data.

## Pre-publication history

The pre-publication history for this paper can be accessed here:

http://www.biomedcentral.com/1471-2288/12/52/prepub
